# Binaural fusion and mis-identification of spatially separated concurrent vowels by cochlear implant users

**DOI:** 10.1121/10.0044921

**Published:** 2026-08-14

**Authors:** Kayla Pierre, Nicole Dean, Holden Sanders, Michelle R. Molis, Lina A. J. Reiss

**Affiliations:** 1 Oregon Hearing Research Center, Oregon Health and Science University, Portland, Oregon 97239, USA; 2 National Center for Rehabilitative Auditory Research, Portland Veterans Administration, Portland, Oregon 97239, USA

## Abstract

Excess binaural fusion can be problematic for listeners in noisy environments, leading to fusion of speech from multiple talkers. Previous research found that hearing aid users experience broad binaural fusion, fusing concurrent dichotic vowels with voice pitch differences of up to an octave [Reiss and Molis (2021) J. Assoc. Res. Otolaryngol. **22**(4), 443–461]. This study examined whether cochlear implant (CI) users, compared to normal-hearing listeners, also exhibit abnormal fusion of concurrent double vowels in free-field conditions. The results revealed that CI users often fuse and mis-identify vowels even with large pitch differences, suggesting that they face additional challenges in noisy environments due to excessive binaural fusion.

## 1. Introduction

Speech perception in noisy environments can be challenging, as listeners must separate overlapping sounds from multiple talkers as part of a process called auditory scene analysis (Bronkhorst, 2000). Auditory scene analysis involves both grouping of sound components likely to arise from the same sound source, and segregation of components likely to arise from different sound sources, using cues such as similarity of frequency, fundamental frequency (*F*0), and spatial location (Bregman, 1990).

Binaural fusion refers to the phenomenon where two separate sounds presented simultaneously to different ears are perceived as a single sound and can be considered a subcategory of grouping. As shown for grouping, listeners with normal hearing (NH) will only binaurally fuse pure tones that evoke similar pitches within 0.1–0.2 octaves (van den Brink, 1976; Reiss *et al.*, 2017), Listeners with hearing loss experience abnormally broad binaural fusion, in which dichotic tones differing by as much as 3–4 octaves in frequency are fused (Reiss *et al.*, 2017) and averaged in pitch (Oh and Reiss, 2017). This broad fusion is also associated with fusion of speech sounds such as vowels, even across large *F*0 differences (Reiss and Molis, 2021). In realistic listening environments, broad binaural fusion predicts less release from masking for *F*0 differences, and research participants report speech from multiple voices blending together (Oh *et al.*, 2022). Thus, while binaural fusion is crucial for sound localization, excessive binaural fusion can pose significant challenges for speech perception, particularly in noisy environments, where speech from multiple talkers may be inappropriately fused and grouped together; In these cases, distinct auditory inputs cannot be adequately separated based on the *F*0 cues typically used for segregation, leading to a blending of sounds that would otherwise be differentiated.

**Fig. 1.**
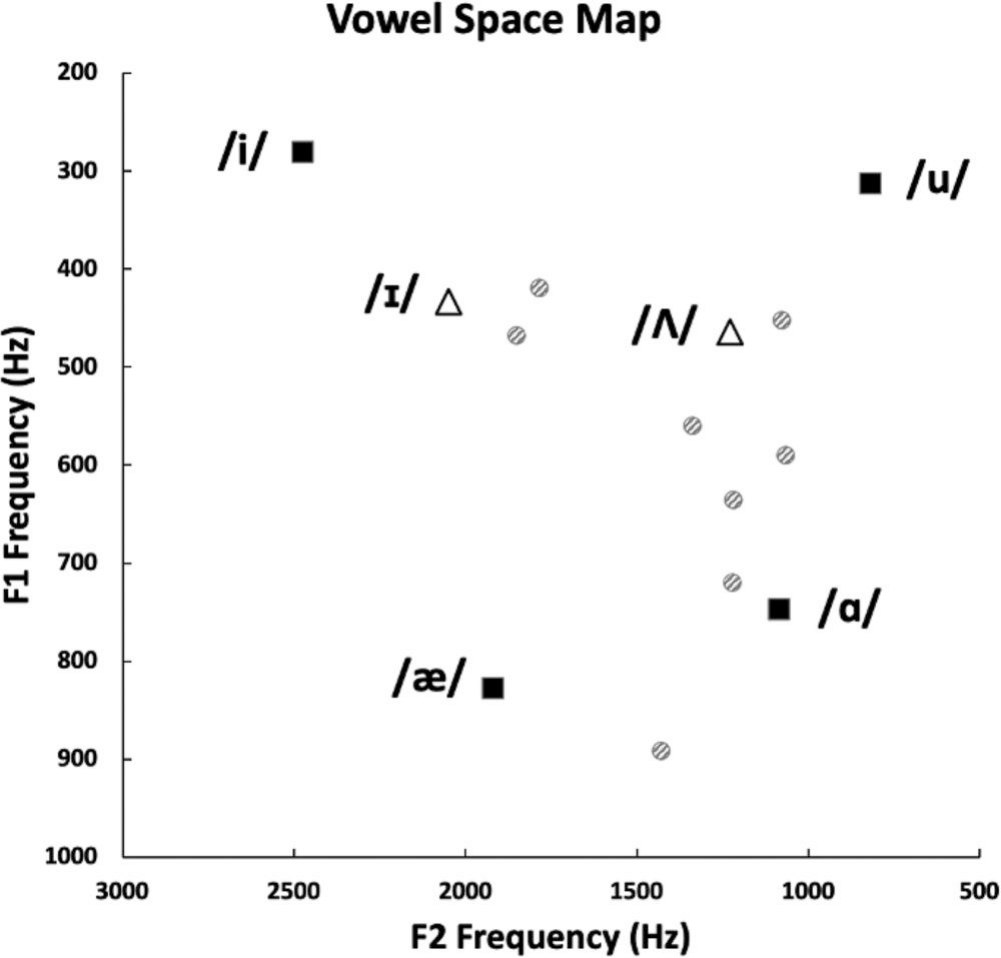
Vowel space map, displaying the first formant frequency (*F*1) on the vertical axis and the second formant frequency (*F*2) on the horizontal axis. Each labeled point on the map corresponds to a vowel sound included as a response option with the solid squares representing the four vowels presented in both training and in the concurrent vowel test, and the open triangles showing the two vowels only presented during training. The small, hatched circles indicate the formant frequencies of the catch stimuli presented only during testing.

Concurrent vowels can be used to assess how broad binaural fusion affects speech segregation. Vowels are useful stimuli for studying spectral integration percepts due to their composition of multiple harmonics of a single *F*0 and multiple peak formants. Each vowel occupies a different region in the vowel space map (Hillenbrand *et al.*, 1995), with some exemplars used in this study shown in Fig. 1.

These regions may overlap, but within them, certain areas represent clearer or more prototypical examples of each vowel sound. Reiss and Molis (2021) demonstrated that in hearing aid (HA) users, broad binaural fusion correlates with difficulties in separating and correctly identifying two different vowels presented dichotically and concurrently over headphones, even with *F*0s differing by as much as 11 semitones. In other words, listeners with broad fusion tend to fuse and misidentify vowels with different voice pitches more frequently than those with narrower fusion ranges.

Molis *et al.* (2026) replicated the findings of Reiss and Molis (2021) in a free-field listening environment, with two vowels presented concurrently over two loudspeakers with ±60 degrees of separation. This setup more accurately reflects real-world listening conditions, where sound localization and environmental acoustics play a significant role in auditory perception. The results of that study showed that these difficulties with concurrent vowel segregation and identification persist in natural listening situations. However, both of these studies involved hearing-impaired listeners without cochlear implants (CIs). Similar to HA users, both bimodal (BM) and bilateral (BL) CI users often experience broad binaural pitch fusion and averaging (Reiss *et al.*, 2014; Reiss *et al.*, 2018). Hence, listeners with CIs and broad fusion may also have similar difficulties with vowel segregation and identification.

To build on this research, the current study investigates whether similar patterns of vowel misidentification occur in CI users in a free-field listening environment. Understanding how CI users process speech in these settings can provide valuable insights into improving assistive listening devices and strategies for this population.

Unlike HAs, which amplify sound, CIs bypass damaged parts of the ear and directly stimulate the auditory nerve via a limited number of electrodes, providing a different sensory experience. BM CI users, who use a CI in one ear together with a HA in the other, have access to both higher resolution acoustic hearing and more sensitive electric hearing. Thus, BM CI users can benefit from the combination of these modalities, particularly for discrimination of musical and voice pitch and in challenging listening environments such as speech in noise (Dorman *et al.*, 2008). In contrast, BL CI users do not have access to higher resolution acoustic hearing. The higher resolution acoustic hearing leads to benefits for music and sound quality in BM CI users (Sucher and McDermott, 2009), as well as benefits for speech perception in noise (e.g., Oh *et al.*, 2023; Visram *et al.*, 2012).

The primary goal of this study was to determine if CI users showed similarly broad vowel fusion and difficulty identifying two vowels in the free field, similar to HA users. The secondary goal was to determine whether the amount of vowel fusion correlates with tone/electrode fusion.

## 2. Methods

### 2.1 Subjects

A total of twenty (*N* = 20) adults participated in the study, ten adult CI users (six females) and ten normal-hearing (NH) adults (nine females). This study was conducted according to the guidelines for the protection of human subjects as set forth by the Institutional Review Board (IRB) of Oregon Health & Sciences University (OHSU), and the methods employed were approved by that IRB. Demographic information and hearing history are shown in Table 1. All subjects were screened for normal cognitive function using the Mini Mental Status Examination (Folstein *et al.*, 1975) with a minimum score of 26 out of 30 required to qualify (Souza *et al.*, 2007); all subjects had scores of 29 or 30. Tympanometry was also conducted for all subjects to verify normal middle ear function.

**Table 1.** Subject demographics. Asterisks (*) indicate programming was altered, and directional microphones were turned off. Processor indicates what CI or HA processor was worn during testing. Duration is how many years the participant has been implanted/worn HAs for each ear. The % correct columns indicate how well the participant did on the training tasks in the monaural and binaural conditions.

CI users								
Participant ID	Sex	Age (years)	Age at onset of deafness (years)	Processor (L, R)	Duration (years: L; R.)	Left (% correct)	Right (% correct)	Both (% correct)
**BL24**	F	65	20	N8, N8	13; 7	84.7	85.4	88.9
**BL30**	F	65	32	N7^*^, N7^*^	12; 12	70.3	83.3	77.7
**BL38**	M	61	5.5	N7^*^, N7^*^	10; 17	65.3	79.9	61.1
**BL48**	M	56	45	N7, N7	20; 20	47.7	57.6	40.2
**BL49**	F	67	9	N7, N8	7; 5	91.7	83.3	91.7
**BM85**	M	39	0	N7^*^, Resound GN	15; 8	43.1	79.9	70.8
**BM102**	M	71	40	N7, Resound GN	2; 6	38.2	85.4	82.6
**BM116**	F	69	35	N7^*^, Resound GN	7; 14	20.8	53.5	62.5
**BM123**	F	60	10	N7, Resound Linx	23; 2	84.0	75.0	97.2
**BM128**	F	63	45	Rondo 2, Resound GN	1; 5	48.6	52.8	56.3

Normal hearing listeners					
Participant ID	Sex	Age (years)	Left (% correct)	Right (% correct)	Both (% correct)
**NH246**	F	27	97.2	97.9	97.2
**NH247**	F	33	98.6	100.0	100.0
**NH248**	M	30	99.3	99.3	98.6
**NH249**	F	24	99.3	100.0	98.6
**NH294**	F	28	99.3	97.9	98.2
**NH300**	F	22	97.9	100.0	95.8
**NH301**	F	28	98.6	99.3	96.5
**NH302**	F	44	96.5	97.2	98.6
**NH306**	F	51	96.5	95.1	94.5
**NH307**	F	55	95.14	99.305	93.75

The NH subjects ranged in age from 24 to 55 years of age [mean and standard deviation (std) = 34.2 ± 11.0 years]. All NH subjects were screened for audiometric thresholds within normal limits [thresholds ≤20 dB hearing level (HL) from 250 to 4000 Hz]. Thresholds were only screened up to 4000 Hz because all testing was conducted with tones and relevant vowel *F*1 and *F*2 information below 4000 Hz.

The CI subjects ranged in age from 39 to 71 years (mean and std = 61.6 ± 8.62 years). Five were BM CI users and five were BL CI users; nine used Cochlear brand implants (Cochlear Ltd., Sydney, Australia) and one (BM128) used Med-El implants (Med-El, Innsbruck, Austria). Duration of hearing device usage ranged from 1 to 23 years (mean and std = 9.67 ± 6.13). There is a significant age difference between NH and CI groups (*t* = 2.11, *p* < 0.001). Because the age range within each group was restricted, correlations of fusion range with age were not evaluated within groups. None of the BM participants had frequency lowering or compression in their HAs.

Subjects were all native American English speakers and were screened on single-vowel perception for both ears (procedure described in Sec. 2.2; results in Table 1). Most of the NH subjects grew up on the East Coast, with the exception of NH246 who grew up in California. The BM and BL subjects came from more diverse locations: BL30 and BM128 grew up in the New York and New Jersey area; BM102 was from Florida; BL48 was from Colorado; BL24, BL38, BL49, BM85, BM116, and BM123 were from the Pacific Northwest or California.

The pure tone averages of the acoustic ears of BM CI participants varied across ears, ranging from 25 to 75 dB HL, with a mean of 34 dB HL.

### 2.2 Stimuli and Procedures

All testing took place in a double-walled sound attenuated booth. To ensure consistent testing conditions across subjects, CI programming was standardized to test all subjects with directional microphones disabled. A subset of subjects, BM85 and BL48, had directional microphones enabled in their everyday maps, which were disabled during testing. BM85 was also tested with and without directional microphones to evaluate the effects of this configuration, but no significant changes were seen.

Vowel fusion and identification testing: Subjects were seated with two Avantone Active Mixcube loudspeakers (Tallman, New York) placed symmetrically at ear height and ±60 degrees azimuth at a distance of 38 inches and calibrated to present vowel stimuli at 65 dBa.

The stimuli were six exemplar synthetic vowels /a/, /ae/, /i/, /Λ/, /I/, and /u/ of 240-ms duration generated with a Klatt speech synthesizer implemented through Praat (Boersma and Weenink, 2016), using a 44.1-kHz sampling rate, and low-pass filtered at 5 kHz. Onset and offset ramps were 20 ms. The *F*1 and *F*2 values of these vowels are shown in Fig. 1 and differ from those used in the original study (Reiss and Molis, 2021). The formant frequencies used in the previous study were constrained to maximally share the *F*1 and *F*2 values of the four corner vowels while maintaining reasonable identifiability. The formant frequencies in the current study were chosen to give more natural-sounding vowel percepts and to enhance vowel distinctiveness given the expanded response set (Hillenbrand *et al.*, 1995; Wright and Souza, 2012). *F*3 was varied to improve vowel quality and *F*4 was fixed at 3500 Hz; all formant values are shown in Table 2. In addition, eight non-exemplar catch stimuli were included, with spectra falling among the exemplar vowels to simulate an averaged percept often experienced with fusion and averaging of the exemplars (Reiss and Molis, 2021). Catch stimuli consisted of a single, potentially ambiguous vowel (hatched circles in Fig. 1) presented from both loudspeakers. These catch stimuli were included to confirm that listeners were not inferring the presence of two vowels from ambiguous percepts produced by the experimental stimuli, i.e., not selecting two vowels when only one catch stimulus was presented. All stimuli were generated at three different non-harmonically related fundamental frequencies of *F*0 = 106.9, 151.2, and 201.8 Hz. These large *F*0 differences were previously shown to be easily segregated by NH listeners with narrow binaural fusion (Reiss and Molis, 2021), but not hearing-impaired listeners with broad binaural fusion.

**Table 2.** Synthetic vowel parameters. Synthetic vowel parameters: vowel identity, label used on the touchscreen, example word containing that vowel, and the four formant frequencies used to synthesize each vowel. Bolded vowels indicate the actual vowels played during both training and testing, the remaining two were only played during training.

Vowel	Screen label	Example word	Formant frequency (Hz)			
			*F*1	*F*2	*F*3	*F*4
**/a/**	**AH**	**HOT**	747	1086.5	2576	3500
**/ae/**	**AE**	**HAD**	828	1920	2620	
**/i/**	**EE**	**HEED**	279.9	2474.9	3076	
**/u/**	**OO**	**HOOT**	312.3	818.5	2365	
/Λ/	UH	HUT	612.4	1295.5	2454.7	
/I/	IH	HID	433	2049	2600	

Prior to testing, subjects were familiarized with the six exemplar vowels under single-vowel presentation using feedback, with each stimulus presented at 3 *F*0 × 4 repeats for a total of 72 stimuli per block. During monaural testing, the contralateral CI/HA was powered off or removed to ensure true monaural input. Subjects were then screened for their ability to perceive the vowels correctly without feedback. NH listeners were screened for monaural vowel identification scores of at least 85% correct; identification scores for CI listeners are shown in Table 1, with the lowest better ear score at 50%. Chance is 16.7%; note that one participant performed near chance for their worse ear (BM116).

During testing, pairs of vowels were presented concurrently, i.e., two vowels were presented simultaneously through the loudspeakers with one vowel presented from each loudspeaker. Two types of stimuli were presented: exemplar vowels and catch stimuli. Although participants were familiarized with all six exemplar vowels during training, only the four corner vowels bolded in Table 2 and shown as filled squares in Fig. 1 (/a/, /ae/, /i/, and /u/) were used during testing. These corner vowels were used because they occupy the extremes of the vowel space and allow for observation of intermediate vowel percepts that may arise from binaural fusion and spectral average as shown previously in Reiss and Molis (2021). Exemplar vowels always differed across the pair presented concurrently (with six unique vowel pairs and the same six vowel pairs also reversed across loudspeakers, giving a total of 12 unique vowel pair-loudspeaker combinations). *F*0 could be the same or different across ears (with three same *F*0 pairs and four different *F*0 pairs consisting of the lowest *F*0 in the left with the middle *F*0 or highest *F*0 in the right, and reversed, for a total of seven *F*0 combinations). This gave a total of 7 *F*0 combinations × 12 vowel pair combinations = 84 exemplar vowel pairs per block. *F*or the catch stimuli, the same stimuli were presented at the same *F*0 across loudspeakers, for a total of 3 *F*0 combinations × 8 catch stimuli = 24 catch trials per block. This led to a total of 108 stimuli per block. Each block was repeated five times for a total of ten repetitions of each vowel pair and *F*0 combination, and five repetitions of each catch stimulus and *F*0. Stimulus presentation was randomized within each block.

On each trial, subjects were instructed to respond on a touchscreen with what they heard. Note that even though only four exemplar vowels were being presented, the touchscreen layout always showed all six options, allowing for the possibility of vowel fusion and averaging to yield vowel percepts not among the four exemplar vowels presented. Participants were told they may hear one or two vowels. Subjects were not informed that the two vowels could differ across loudspeakers or that not all vowels were presented. Listeners had the option to choose one vowel if they heard one vowel, or two vowels if they heard two different vowels; a “Done” option allowed listeners to indicate when they had finished with either one or two vowel choices. No feedback was provided.

Percentage of single vowel responses and percentage of vowels correctly identified were calculated only from exemplar vowel trials. Percentage of single vowel responses were calculated as: (number of single vowel responses from exemplar trials)/(total number of exemplar vowel trials) × 100. Vowel identification was calculated as the percentage of trials on which both vowels were correctly identified: (number of trials with both exemplar vowels correct)/(total number of exemplar vowel trials) × 100, with total number of trials including both one-vowel and two-vowel responses. Catch trials were used as a check for double vowel responses indicating that subjects were inferring and reporting two vowels instead of reporting the single vowel actually heard.

Tone/electrode fusion range measurements: For tone/electrode fusion range measurements, BM CI users were presented with acoustic tones via headphones to the acoustic ear, while electric pulse trains were delivered via direct connection to the CI ear (Reiss *et al.*, 2014). For BL CI users, electrical pulse trains were sent to both devices/ears (Reiss *et al.*, 2018). Briefly, stimuli were presented simultaneously across ears, with the reference electrode fixed (electrode 18 for Cochlear America devices and electrode 5 for Med-El devices) and the contralateral stimulus varied across trials. In each trial, a comparison tone or electric pulse train was delivered to the contralateral ear, with both stimuli of 1500-ms duration. On each trial, the subject was asked to indicate whether a single fused tone or two different tones were heard. Fusion range was calculated as the range of frequencies or electrodes over which the comparison stimuli fused with the reference over 50% of the time. For BL CI users, the fusion range in units of electrodes was converted to millimeters based on the known interelectrode distances then converted to octaves based on the approximate relationship of frequencies of the cochlear distances (Greenwood, 1990; Hartling *et al*, 2020).

### 2.3 Statistical analysis

For these analyses, all percent single-vowel responses and percent correct scores were transformed into rationalized arcsine units (Studebaker, 1985). Two repeated-measures analyses of variance were performed with Δ*F*0 (0, 6, or 11 semitones) as a within-subjects factor and group (NH, CI) as a between-subjects factor. *Post hoc* comparisons were conducted when significant effects were found and were corrected for multiple comparisons using a Bonferroni correction. The BL and BM groups were initially analyzed separately, but due to the small sample size and similarity of trends between the two groups, they were ultimately combined into a single CI group. The assumption of sphericity was rejected by Mauchly's test (Mauchly, 1940), hence Greenhouse-Geisser corrections (Greenhouse and Geisser, 1959) were applied to analyses where appropriate.

## 3. Results

### 3.1 Vowel fusion changes with Δ*F*0

On trials where two vowels were presented, a single-vowel response indicates that subjects only heard one vowel and thus experienced fusion of two different vowels across the ears into a single percept. Thus, the percent of single vowel responses is taken to indicate the amount of vowel fusion. For NH listeners, vowel fusion decreased from ∼75% to under 30% with increases in Δ*F*0. CI users showed more variable changes in vowel fusion as Δ*F*0 increased. One CI user (BL123) showed comparable decreases, four CI users showed moderate decreases (BL24, BL49, BM123, BM128), and all remaining CI users showed little to no change. In other words, as pitch differences increased, NH listeners showed a marked reduction in single vowel responses, indicating successful segregation of the two vowel sounds; whereas almost all CI users exhibited clear difficulty separating the vowels from one another. One CI user, BM85, differed from all other subjects in responding 45% of the time that they heard two sounds to the catch stimuli, compared to <5% of the time for other NH and CI subjects, suggesting that this participant may have responded based on some other criterion than truly hearing two sounds. This may explain why their response did not change much with fundamental frequency difference.

**Fig. 2.**
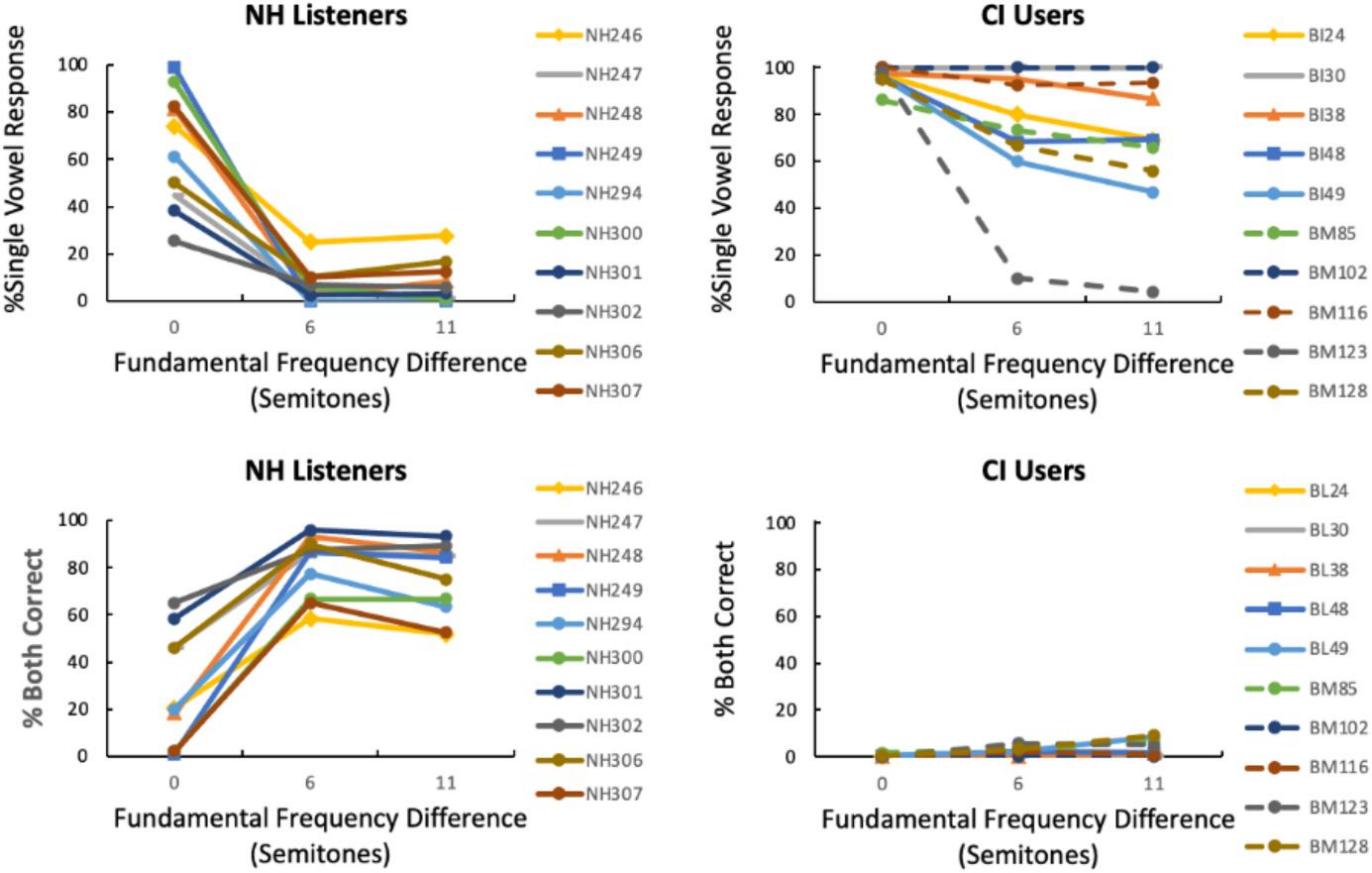
Top row: Percent of single vowel responses plotted as a function of the pitch difference Δ*F*0 for NH listeners (left) and CI users (right). Bottom row: Percent correct identification of two vowels plotted as a function of the pitch difference Δ*F*0 for NH listeners (left) and CI users (right). BM, bimodal CI/HA users; BL, bilateral CI users.

For percent single vowel responses, there were significant main effects of Δ*F*0 (*F*(1.07, 11.65) = 47.08, *p* < 0.001), and group (*F*(1, 18) = 53.01, *p* < 0.001), and a significant interaction observed between Δ*F*0 and group (*F*(1.07, 19.18) = 6.08, *p* < 0.05). For the overall effect of Δ*F*0, *post hoc* tests showed significant differences between Δ*F*0 = 0 and Δ*F*0 = 6 (*p* < 0.001) but not between Δ*F*0 = 6 and Δ*F*0 = 11(*p* = 0.08). *Post hoc* tests also showed significant interactions between group and Δ*F*0 = 0 vs Δ*F*0 = 6 (*p* < 0.001) and Δ*F*0 = 6 vs Δ*F*0 = (*p* < 0.05). As can be seen in the top row of Fig. 2, this interaction is due to the different trends of CI users from NH listeners with increases in Δ*F*0, including the continued reduction in single vowel responses as Δ*F*0 increases from 6 to 11 semitones seen for CI listeners but not NH listeners.

### 3.2 Vowel identification changes with Δ*F*0

While NH listeners demonstrated improved vowel identification with increased Δ*F*0, CI users did not exhibit significant improvement (Fig. 2, bottom row). In other words, as the pitch difference increased, NH listeners were able to identify as well as segregate both vowels correctly. In contrast, CI listeners did not improve in vowel identification as pitch differences increased. Percent correct for at least one vowel was computed and compared with better-ear monaural vowel identification (Fig. 1 in the supplementary material); no significant correlation was seen. Even CI users with high monaural scores showed low double-vowel identification, confirming effects were not purely monaural. Even CI users who showed reduced vowel fusion with increased Δ*F*0, such as BM123, had limited improvement in vowel identification compared to NH listeners.

For percent correct performance on selecting both vowels correctly, there was a significant main effect of Δ*F*0 (*F*(1.24, 22.29) = 63.16, *p* < 0.001) and group (*F*(1, 18) = 149.06, *p* < 0.001) and a significant interaction observed between Δ*F*0 and group (*F*(1.24, 22.29) = 31.75, *p* <0.001). *Post hoc* tests of the overall effect of Δ*F*0 showed significant differences between Δ*F*0 = 0 and Δ*F*0 = 6 (*p* < 0.001) but not between Δ*F*0 = 6 and Δ*F*0 = 11 (*p* = 0.21). Once again, *post hoc* tests also showed significant interactions between group and Δ*F*0 = 0 vs Δ*F*0 = 6 (*p* < 0.001) and Δ*F*0 = 6 vs Δ*F*0 = (*p* < 0.005). As can be seen in the bottom row of Fig. 2, this interaction is due to the different trends of CI users from NH listeners with increases in Δ*F*0, such as the minimal improvement in percent correct vowel identification for Δ*F*0 > 0.

### 3.3 Confusion matrices show spectral averaging

**Fig. 3.**
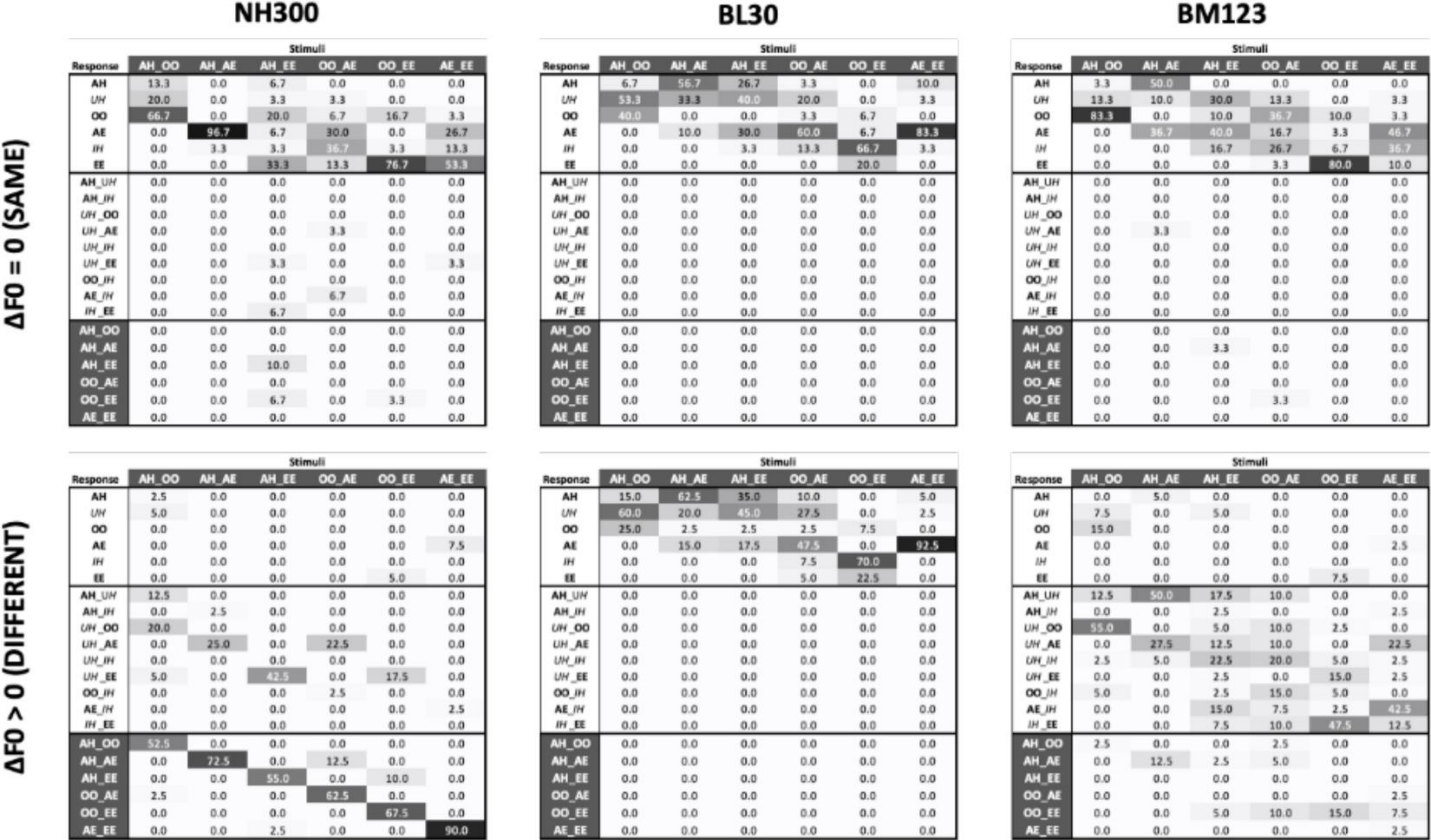
Confusion matrices of three subjects (NH300, BL30, and BM123) for Δ*F*0 = 0 semitones (top) and Δ*F*0 > 0 semitones (bottom). Vowel response options are shown along the left most column, and the combinations of vowel pairs presented are shown along the top row. Within this chart are the subject responses. Responses along the top six rows represent when vowels were fused, and single vowel responses were selected. Responses along the middle nine rows indicate selection of double vowel responses with one vowel not from the actual set presented. Responses along the bottom six rows indicate double vowel responses with both vowels from the set presented, with responses along the diagonal indicating how many times the correct pair was chosen.

Confusion matrices were generated in order to visualize which vowels were perceived when vowels were fused or incorrectly identified (Fig. 3). Each column represents the stimulus pair presented, and each row represents the responses given. The top six rows of each confusion matrix show the single vowel responses, and the middle and bottom rows show the two-vowel responses; the bottom six rows show the actual vowel pair combinations possible from the four exemplar vowels presented, with correct responses on the diagonal.

Consistent with the data shown in Fig. 2, when Δ*F*0 = 0, both NH and CI users frequently fused the vowels, but the confusion matrices reveal additional details beyond percent correct or single vowel responses. Specifically, the fused vowels were often misidentified as a new vowel, one not present in the actual stimulus, suggesting spectral averaging rather than simple errors in identification. For instance, when the vowel pair /a/-/i/ (AH-EE) was presented, across both groups the vowel /ʌ/ (UH) was often selected despite it not being in the original pair. Another example includes the presentation of /ae/-/u/ (AE-OO), where both groups commonly identified the vowel /I/ (IH), reflecting a perceived blending of the spectral characteristics of the original pair. For Δ*F*0 > 0, NH listeners more frequently identified both vowels, and these identifications were often correct, as indicated by the higher frequency of responses along the diagonal in the confusion matrix. In contrast, CI users continued to show high rates of fusion, and their misidentifications persisted, with the confusion matrices showing that the new vowels perceived when one vowel was identified often deviated significantly from the actual vowels presented, likely due to their perceived spectral profiles resembling an average of the presented vowels.

## 4. Discussion

The findings indicate that CI users experience excessive binaural fusion and difficulty segregating two vowels with differing *F*0s in free-field listening conditions. As Δ*F*0 increased, NH listeners were able to hear and identify two vowels more accurately, while CI users predominantly perceived a single vowel and misidentified the vowels even when they perceived two. These results replicate patterns observed in HA users both over headphones and in the free field (Reiss and Molis, 2021; Molis *et al.*, 2026) and demonstrate for the first time that CI users show broad binaural fusion of vowels across large voice-pitch differences of up to 11 semitones, i.e., across *F*0s of male and female voices.

Fusion ranges in the CI users in this study were similar to those reported previously, within the 0–5 octave range for BM CI users and the 0–2.3 octave range for BL CI users (Reiss *et al.*, 2014, 2018; Hartling *et al.*, 2020). The sample size was small, which limited the power for correlation analyses to show significant relationships. More data are needed before an association of fusion range with vowel fusion can be ruled out in CI users.

There were some methodological differences from Reiss and Molis (2021). In addition to presentation in the free field, vowels were synthesized to be more natural sounding, and more vowel options were given (six instead of four). However, the overall findings were robustly similar to those observed previously. The only difference in results was the finding of different averaged vowel percepts as shown by the confusion matrices. These different vowel percepts may reflect either the different *F*1 and *F*2 values of the vowels presented or the availability of more vowel response options.

Overall, the results suggest that similarly to HA users, the challenges faced by CI users extend beyond monaural limitations and are likely due to alterations in binaural processing. These findings suggest that excessive binaural fusion underlies an impairment in segregation of talkers, particularly segregation based on *F*0 differences, regardless of hearing device type. Further, this occurred in the free field, extending that this issue extends to real-world listening environments. This impairment underscores the need for tailored auditory training and technological interventions, such as directional microphones that can specifically address these challenges.

## Supplementary Material

See supplementary material for figure showing relationship between the percent single vowel response and the fusion range.

## Data Availability

The data that support the findings of this study are available from the corresponding author upon reasonable request.
